# The incidence of acute pulmonary embolism following syncope in anticoagulant-naïve patients: A retrospective cohort study

**DOI:** 10.1371/journal.pone.0193725

**Published:** 2018-03-07

**Authors:** Danny Epstein, Gidon Berger, Noam Barda, Erez Marcusohn, Yuval Barak-Corren, Khitam Muhsen, Ran D. Balicer, Zaher S. Azzam

**Affiliations:** 1 Department of Internal Medicine "B", Rambam Health Care Campus, Haifa, Israel; 2 Division of Pulmonary Medicine, Rambam Health Care Campus, Haifa, Israel; 3 Clalit Research Institute, Chief Physician’s Office, Clalit Health Services, Tel Aviv, Israel; 4 Predictive Medicine Group, Boston Children’s Hospital, Boston, United States of America; 5 Shaare Tzedek Medical Center, Jerusalem, Israel; 6 Department of Epidemiology and Preventive Medicine, School of Public Health, Sackler Faculty of Medicine, Tel Aviv University, Tel Aviv, Israel; 7 Epidemiology Department, Ben Gurion University of the Negev, Be’er Sheba, Israel; 8 The Rappaport's Faculty of Medicine, The Technion Institute, Haifa, Israel; National and Kapodistrian University of Athens, GREECE

## Abstract

**Background:**

A recently published, large prospective study showed unexpectedly high prevalence of acute pulmonary embolism (APE) among patients hospitalized for syncope. In such a case, a high incidence of recurrent pulmonary embolism is expected among patients who were discharged without APE workup.

**Objectives:**

To determine the incidence of symptomatic APE among patients hospitalized for a first episode of syncope and discharged without APE workup or anticoagulation.

**Methods:**

This retrospective cohort study included patients hospitalized at Rambam Health Care Campus between January 2006 and February 2017 with a primary admission diagnosis of syncope, who were not investigated for APE and were not taking anticoagulants. The patients were followed up for up to three years after discharge. The occurrence of venous thromboembolism (VTE) during the follow-up period was documented.

**Results:**

The median follow-up duration was 33 months. 1,126 subjects completed a three-year follow-up. During this period, 38 patients (3.38%) developed VTE, 17 (1.51%) of them had APE. The cumulative incidence of VTE and APE was 1.9% (95% CI 1.3%-2.5%) and 0.9% (95% CI 0.4%-1.3%) respectively. Only seven subjects developed APE during the first year of follow-up. The median times from the event of syncope to the development of APE and VTE were 18 and 19 months respectively.

**Conclusions:**

The cumulative incidence of VTE during a three-year follow-up period after an episode of syncope is low. In the absence of clinical suspicion, a routine diagnostic workup for APE in patients with syncope cannot be recommended.

## Introduction

Syncope is a common symptom accounting for 1–3% of emergency department visits and 6% of hospital admissions in the U.S.[1]. Various benign to life-threatening clinical conditions can cause syncope [2]. However, in 30–50% of cases the etiology for syncope remains unknown even after appropriate clinical workup[2–4].

The association between syncope and acute pulmonary embolism (APE) has been questioned for many decades. Syncope has been found to be the initial or predominant clinical feature in 5.5–13% of patients with APE[5–7]. Most of these patients present with a typical clinical picture of APE[5,7]. Accordingly, current guidelines consider isolated syncope as an infrequent initial manifestation of APE and do not routinely recommend a diagnostic workup for this etiology[8]. Recently, however, Prandoni et al.[9] challenged this paradigm, showing that nearly 17% of all patients presenting with a first episode of syncope, and who underwent systematic workup for APE, were positive for the disease. The authors showed that among patients with an alternative explanation for syncope, 12.7% had a concomitant diagnosis of APE. Among the patients without an established cause for syncope, 25% were diagnosed with APE following workup[9].

Anticoagulation therapy is the cornerstone of APE management; its major role is to prevent APE recurrence[10]. In patients taking anticoagulants for at least 90 days following the diagnosis of APE, the risk of recurrence is greatest in the first two weeks following diagnosis.[11]. Recurrence rates of venous thromboembolism (VTE), which encompasses both APE and deep vein thrombosis, are 10%, 12.9% and 16.5% during the first six months, one year and two years, respectively[12,13]. In untreated patients, the recurrence rate is expected to be even higher.

While Prandoni et al.’s observations and their clinical implications still need to be confirmed in additional populations, they suggest that a substantial portion of patients presenting with syncope have APE. Based on this hypothesis, we postulated that some patients with syncope, and who were not investigated for APE (based on lack of clinical suspicion) and not anticoagulated, might be diagnosed with APE after discharge.

In this study we aimed to outline the natural history of anticoagulant-naïve patients with no clinical signs of VTE hospitalized for a first episode of syncope with respect to the occurrence of VTE and to determine the incidence rates of symptomatic APE in this population.

## Materials and methods

### Study population and design

A retrospective cohort study was conducted with the use of medical records of hospitalized adult patients who were admitted following syncope episode to the Rambam Health Care Campus (RHCC) in Haifa, Israel between January 1, 2006 and February 4, 2017. The RHCC is a 1,000-bed academic hospital serving over two million residents in northern Israel with 80,000–90,000 hospitalizations annually.

The study population included patients 18 years or older with a primary admission diagnosis of syncope, as mentioned in the admission report of the emergency department (ICD9 code 780.2). The diagnosis code of syncope in administrative registries is highly specific and sensitive[14]. Patients were hospitalized for investigation of syncope due to trauma related to falls, severe coexisting conditions, failure to identify an explanation for the syncope, or a high probability of cardiac syncope. All patients underwent clinically guided, routine syncope workup.

Excluded from the study were patients who died during the index hospitalization, patients who left against medical advice, and patients who were transferred to or from another acute care facility. Patients were also excluded if they received anticoagulants, or underwent APE workup (d-dimer, computed tomographic pulmonary angiography or ventilation–perfusion lung scanning or limb Doppler ultrasound) during the index hospitalization. Patients who were prescribed anticoagulants for any indication (excluding VTE) at discharge or anytime during the follow up period (based on Anatomical Therapeutic Chemical classification system codes for antithrombotic preparations B01A, excluding B01AC) were also excluded at treatment initiation. Patients who were hospitalized for syncope evaluation during the previous two years were excluded as well because we hypothesized that recurrent syncope are unlikely to be caused by VTE as the sole presentation. Same strategy was used by Prandoni and others.[9,15]

### Follow-up

We followed patients who met the inclusion criteria described above and were insured by Clalit Health Services (CHS). Follow-up was performed via linkage with CHS’s computerized database. CHS is the largest health maintenance organization (HMO) in Israel covering over 50% of population, with little turnover and loss to follow-up. The database includes demographic and administrative data, in/out patient data, lab results and imaging results performed in 14 hospitals and over 1,500 clinics. The linkage was performed using personal unique identification number that each Israeli citizen receives at birth. This allowed access to the electronic medical records of patients insured by CHS to ascertain medical outcomes.

Each patient was followed from discharge until the earliest of the following events: occurrence of a VTE outcome, initiation of anticoagulation therapy (for reason other than VTE), death due to reason other than VTE, leaving CHS, or the end of the three-year follow-up period.

### Data collection and definition of the study variables

Data on the index hospitalizations for syncope, based on the specific primary ICD9 code 780.2, were retrieved from Prometheus, RHCC’s integrated electronic medical records system.

Information on the following demographic and clinical characteristics was retrieved from medical records: age (in years), sex (male or female), previous VTE (yes or no), active cancer (yes or no), congestive heart disease (yes or no), diabetes mellitus (yes or no), chronic renal failure (yes or no), ischemic heart disease (yes or no), and valvular heart disease (yes or no). We used Charlson index to assess comorbidity burden. Data on vital signs at presentation were also collected, including heart rate, blood pressure, and oxygen saturation. Ascertainment of the diagnosis of VTE and initiation of anticoagulation subsequent to index hospitalization were performed through CHS’s computerized database. The outcome variables included development of pulmonary embolism (ICD9 code 415.1X) or deep vein thrombosis (ICD9 453.2, 453.3, 453.4X, 453.8X, 453.9). Subjects who died during hospital admission due to VTE were also counted.

The diagnoses were extracted from community and hospital records, as well as from the CHS chronic disease registry. Diagnoses from the community records were further validated by a free text-diagnosis description. This method has been used and validated in previous studies[16].

### Statistical analysis

Baseline characteristics were summarized using descriptive statistics. The Kolmogorov–Smirnov test was used to examine significant deviations from the normal distribution for continuous variables. Student’s *t* test for independent samples was used to compare means of variables with normal distribution, and Mann-Whitney U test was used for variables that deviated from the normal distribution. Chi-squared test and Fisher exact test were used to analyze differences between dichotomous variables, depending on the sample size. P-value<0.05 was considered statistically significant.

Survival was estimated, based on cumulative incidence curves generated by a non-parametric competing risk analysis with the Andersen-Johansen estimator. Estimation standard errors were calculated with the Greenwood formula for a 95% confidence interval.

Data were analyzed with the Statistical Package for the Social Sciences, version 23.0 (IBM, Armonk, New York, USA), R Foundation for Statistical Computing, version 3.4.0 (Vienna, Austria) and Microsoft Excel version 14.0 (Redmond, Washington, US).

### Ethical aspects

The study was approved by the Institutional Review Board at RHCC and CHS. Owing to the retrospective nature of the study, informed consent was waived. This trial is registered with ClinicalTrials.gov (number NCT03034525).

## Results

**Fig 1:**
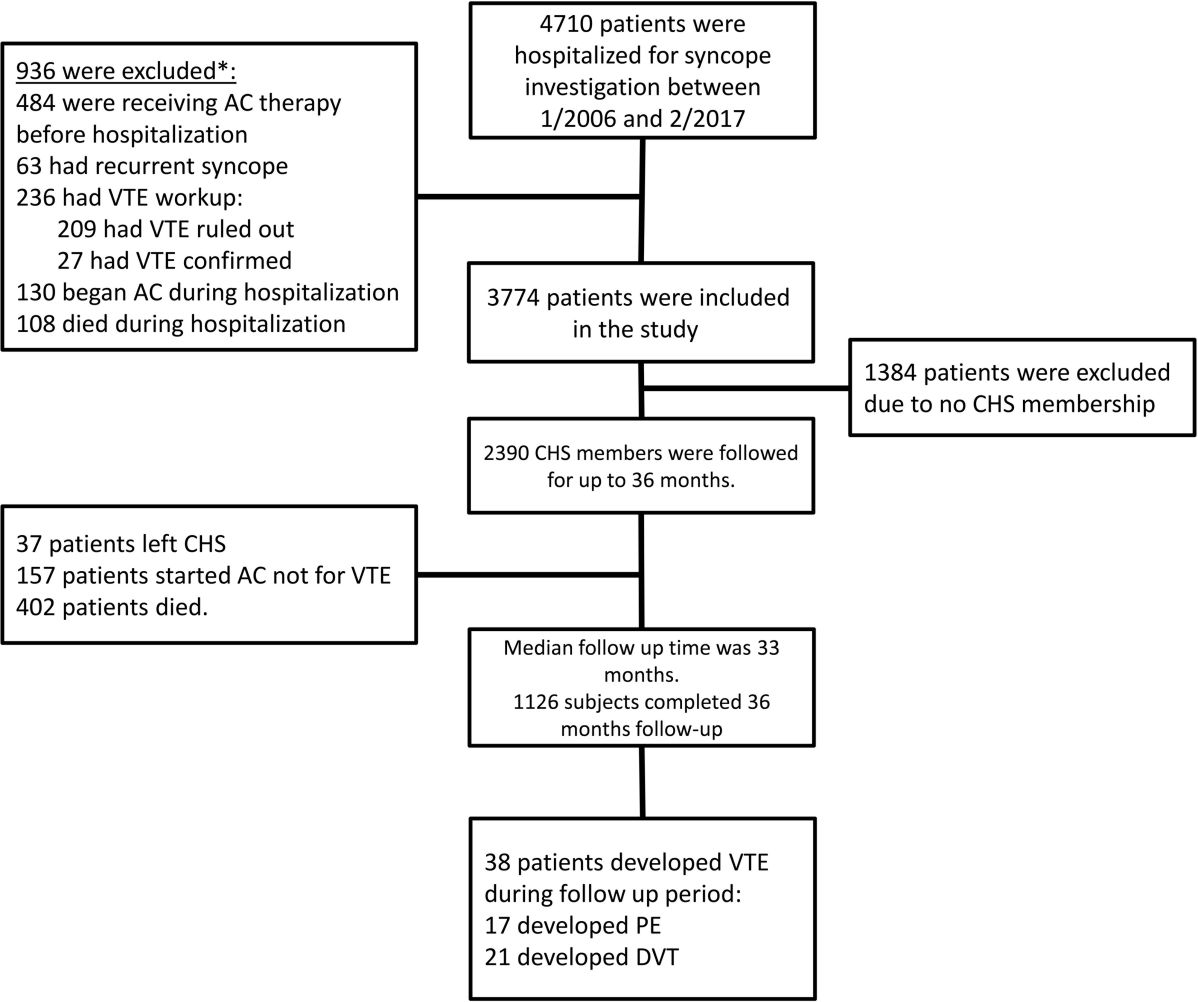
Study flow chart. * Some patients were excluded due to more than one criterion. CHS–Clalit Health Services; AC- anticoagulation; VTE- venous thromboembolism; PE- pulmonary embolism; DVT- deep vein thrombosis.

During the study period, a total of 4,710 patients were admitted to RHCC for investigation of syncope; among them 236 (5%) underwent VTE workup, which was confirmed in 27 cases (0.6% of all patients and 11.4% of patients investigated) as the etiology of syncope. A total of 3,774 (80%) patients met the basic inclusion criteria (Fig 1), of whom 2,390 patients (63%) were insured by CHS and eligible for the current study.

Patients who were enrolled in the study were older than those who were insured in other HMOs and not included in this study: median age 72.5 vs. 63.7 years (p = 0.001), and had significantly more comorbidity (Table 1). However, the groups did not differ regarding sex distribution or previous VTE history (Table 1).

**Table 1:** Baseline demographic and clinical characteristics of patients admitted to Rambam Health Care Campus for investigation of first episode of syncope from January 2006 to February 2017.

Characteristic		All patients (n = 3774)	CHS members (n = 2390)	Insured by other HMOs (n = 1384)	p-value
**Age-years**	**Mean (SD)**	64.8 (20.0)	68.1 (18.3)	59.1 (21.6)	0.001
	**Median (interquartile range)**	69.8 (53.8–80.3)	72.5 (58.3–82)	63.7 (44.6–76.7)	
	**>70yr-no (%)**	1866 (49.4%)	1331 (55.7%)	535 (38.7%)	0.001
	**>80yr-no (%)**	973 (25.8%)	731 (30.6%)	242 (17.5%)	0.001
**Male sex-no (%)**		2131 (56.5%)	1333 (55.8%)	798 (57.7%)	0.28
**Heart rate >100bpm-no (%)**^**1**^		203 (6.2%)	116 (5.7%)	87 (7.1%)	0.1
**Systolic BP <110mmHg-no (%)**^**1**^		322 (9.84%)	186 (9.1%)	136 (11.2%)	0.06
**Oxygen saturation <92% while breathing ambient air- no (%)**^**2**^		92 (2.9%)	69 (3.5%)	23 (2.0%)	0.022
**Median length of hospital stay (interquartile range)-days**		4 (2–6)	4 (2–6)	3 (2–5)	0.001
**Previous VTE**^**3**^**-no (%)**		10 (0.3%)	7 (0.29%)	3 (0.22%)	0.9
**Hypertension-no (%)**		1925 (51%)	1302 (54.5%)	623 (45.0%)	0.001
**Diabetes mellitus-no (%)**		1028 (27.2%)	732 (30.6%)	296 (21.4%)	0.001
**Atrial fibrillation**^**3**^**-no (%)**		307 (8.1%)	220 (9.2%)	87 (6.3%)	0.002
**Chronic renal failure-no (%)**		389 (10.3%)	294 (12.3%)	95 (6.9%)	0.001
**Chronic Obstructive Pulmonary Disease -no (%)**		142 (3.8%)	109 (4.6%)	33 (2.4%)	0.001
**Malignant neoplasm-no (%)**		400 (10.6%)	276 (11.5%)	124 (9.0%)	0.015
**Median Charlson comorbidity index (interquartile range)**		1 (0–3)	2 (0–4)	1 (0–2)	0.001

1- Heart rate and blood pressure at admission was available for 3272 patients (86.7%). 2053 were CHS members (85.9%) and 1220 (88.2%) were members of other HMOs.

2- Oxygen saturation at admission was available for 3160 patients (83.7%). 1994 were Clalit Health Services members (83.4%) and 1160 (83.8%) were members of other HMOs.

3- Atrial fibrillation or venous thromboembolism without anticoagulation treatment.

CHS–Clalit Health Services; HMO—health maintenance organization; SD- standard deviation; bpm- beats per minute; BP- blood pressure; VTE- venous thromboembolism.

The median follow-up duration was 33 months. During the follow-up period, 157 patients (6.6%) initiated anticoagulation for indication other than VTE, 37 (1.6%) discontinued their membership with CHS, and 402 died (16.8%) (Fig 1). During the follow-up period, 38 patients (3.38%) developed VTE and 17 patients (1.51%) developed APE. Seventeen patients (45%) were diagnosed with VTE during the first year of follow-up, nine of them within the first 90 days of discharge. Only three and seven subjects developed APE during the first 90 and 360 days respectively.

**Fig 2:**
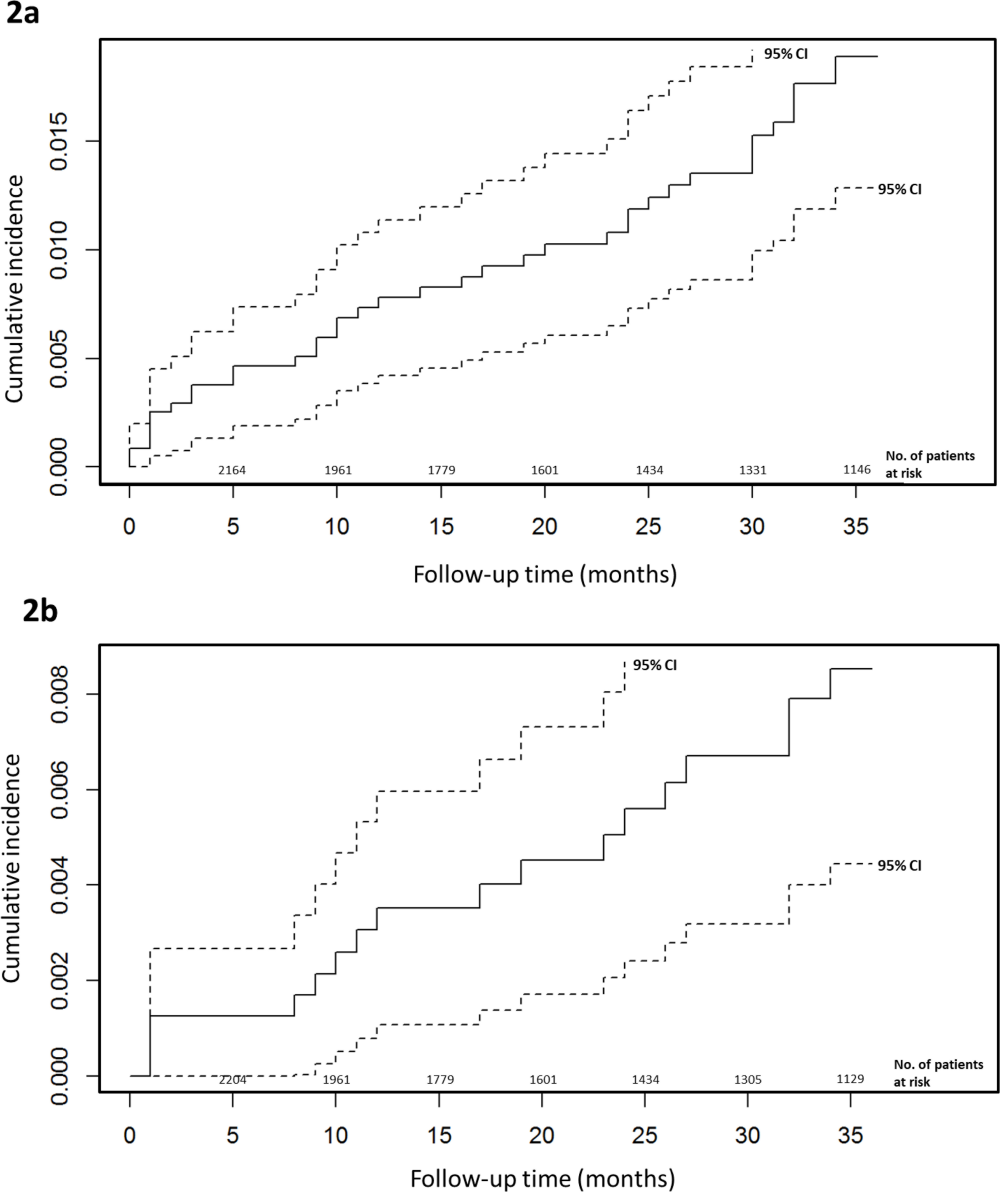
Cumulative incidence with 95% confidence interval estimate of venous thromboembolism (Fig 2A) and acute pulmonary embolism (Fig 2B) during 36 months after hospitalization for investigation of syncope.

Fig 2A and 2B show the cumulative risk of VTE and APE during the 36 months after syncope respectively. Using competing risks-survival analysis, we calculated the cumulative incidence of PE at three months, one year, two and three years as 0.1% (95% CI 0.0%-0.3%), 0.3% (95% CI 0.1%-0.5%), 0.5% (95 CI 0.2%-0.8%) and 0.9% (95% CI 0.4%-1.3%) respectively. The corresponding figures for VTE were 0.3% (95% CI 0.08%-0.5%), 0.7% (95% CI 0.4%-1.1%), 1.1% (95 CI 0.7%-1.5%), and 1.9% (95% CI 1.3%-2.5%) respectively. The median times from hospitalization to development of PE and VTE were 18 and 19 months respectively.

Patients who developed VTE during the follow-up period were significantly older than those who did not (median age 80.2 vs. 72.2, p = 0.027) and had a significantly more frequent history of previous VTE, (7.9% vs. 0.2%, p < 0.001). The former had a higher percentage of patients with heart rate beat > 100 beats per minute and oxygen saturation < 92% at baseline (index hospitalization), but these differences were not significant statistically. There was no significant difference between the groups in the median of Chalrson comorbidity index or history of malignancy (Table 2).

**Table 2:** Comparison of characteristics of patients who developed VTE during the follow-up to those with no VTE diagnosis.

Characteristic		Patients with VTE (n = 38)	Patients without VTE (n = 2352)	p-value
**Age-years**	**Median (interquartile range)**	80.2 (64.3–86.2)	72.2 (58.2–81.9)	0.027
	**Mean (SD)**	74.6 (16.1)	68 (18.2)	
	**>70yr-no (%)**	27 (71.1%)	1304 (55.4%)	
	**>80yr-no (%)**	19 (50.0%)	712 (30.3%)	
**Male sex-no (%)**		16 (42.1%)	1316 (56.0%)	0.12
**Heart rate >100bpm- no (%)**^**1**^		4 (13.3%)	111 (5.5%)	0.15
**Systolic BP <110mmHg- no (%)**^**1**^		3 (10.0%)	182 (9.0%)	0.9
**Oxygen saturation <92% while breathing ambient air-no (%)**^**1**^		3 (10.0%)	66 (3.4%)	0.14
**Previous VTE**^**2**^**-no (%)**		3 (7.9%)	4 (0.2%)	<0.001
**Malignant neoplasm-no (%)**		5 (13.2%)	271 (11.5%)	0.7
**Median Charlson comorbidity index (interquartile range)**		2 (1–6)	2 (0–4)	0.6

1- Heart rate, blood pressure at admission was available for 30 patients (79.0%) in the VTE group and 2023 (86.0%) patients with no VTE. Oxygen saturation at admission was available for 30 patients (79.0%) in VTE group and 1964 (83.5%) patients with no VTE.

2- Venous thromboembolism without anticoagulation treatment.

Out of 17 patients who developed APE during follow- up period, 12 were hospitalized at RHCC and their medical charts were subject to full-chart review. The age range on the index admission for syncope was 69–93 years; a trial fibrillation was recorded in four (33.3%) patients, four (33.3%) had diabetes mellitus, three (25%) had cancer, two had COPD, two patients had ischemic heart disease and one patient had end-stage renal failure (Table 3). APE was diagnosed in five patients (42%) within two months of discharge, while the others developed PE between nine to 33 months following their discharge (Table 3).

**Table 3:** Detailed description of 12 of 17 patients who developed APE subsequent to discharge after first syncope episode during a 3-year follow-up period.

Patient No.	Sex	Age at index admission, years	Major co-morbidities	Reason for syncope index admission	Clinical circumstances of first syncope at index admission	Wells score during syncope admission	Time until developing APE (days/months)	Clinical circumstances of PE
1.	F	84	AF	Syncope, profound sweating, low saturation	No etiology for syncope was identified	3	33.1 months	Hospitalization for dyspnea & DVT signs.
2.	M	87	AF	First traumatic syncope	No etiology for syncope was identified	0	32.1 months	Hospitalization for pneumonia, PE was identified on CTA.
3.	F	73	DM	Syncope, severe anemia	Normal vital sign, ECG &Troponin were normal.	0	27.7 months	Hospitalization for cerebral hemorrhage with complicated clinical course. Asymptomatic PE was identified in chest CTA.
4.	M	69	DM, active cancer	First traumatic syncope	No etiology for syncope was identified	1	25.4 months	Developed bilateral PE after prolonged hospitalization in ICU due to septic shock
5.	F	80	DM	First traumatic syncope	Normal vital signs, ECG & Troponin. No etiology for syncope was identified	0	15.9 months	PE was diagnosed during prolonged & complicated hospitalization due to sepsis secondary to leg gangrene.
6.	M	86	IHD	Syncope after excessive nitrates usage	Hypotensive at admission. Normal ECG &Troponin.	0	12.6 months	Asymptomatic PE was identified during abdominal CTA.
7.	M	93	ESRD, active cancer, AF	First syncope	Normal vital signs, ECG, Troponin, Echo & Holter No etiology for syncope was identified	1	9.1 months	Hospitalization for colonic pseudo-obstruction, during abdominal CTA and developed shortness of breath.
8.	M	75	COPD	First syncope during physical activity	Normal vital signs, ECG, Troponin, Echo, Holter No etiology for syncope was identified	0	2.0 months	Admitted due to chest pain, dyspnea, & desaturation. Bilateral PE was diagnosed.
9.	F	93	-	Syncope, chest pain	Tachycardia Normal ECG & Troponin	1	28 days	Admitted due to recurrent syncope, & DVT signs.
10.	M	90	IHD, AF, metastatic cancer	First syncope during physical activity	Tachycardia at admission. Normal ECG, Troponin, & Holter No etiology for syncope was identified	2.5	14 days	Admitted due to chest pain & dyspnea.
11.	*F*	*91*	*DM*	*Syncope after mild bleeding*.	*Tachycardia*.	*1*.*5*	*13 days*	*Admitted due to dyspnea & DVT signs*.
12.	F	78	COPD	First traumatic syncope	Tachycardia, low saturation	1.5	10 days	Admitted due to dyspnea, large anatomical PE without signs of right ventricle dysfunction was identified by CTA. The patients died during hospitalization.

F- female; M- male; AF- atrial fibrillation; DVT- deep vein thrombosis; PE- pulmonary embolism; CTA- computed tomography angiography; DM- diabetes mellitus; ICU- intensive care unit; IHD- ischemic heart disease; ESRD- end stage renal disease;; COPD- chronic obstructive lung disease.

## Discussion

Prandoni's study has sparked a debate about the true incidence of pulmonary embolism among patients hospitalized with syncope and whether the filling defects observed in computed tomography angiography represent clinically significant APE. Since the major clinical implication of acute PE is recurrent PE, our study followed the natural history of these patients, regarding the occurrence of VTE.

Approximately 40% of the U.S. population will experience a syncopal episode in their lifetime, and 30 to 50% of them will be admitted to a hospital for further evaluation[17]. Despite extensive evaluation and a median hospital cost of $8,500 per admission, the etiology remains unexplained in a significant number of cases[2–4,17]. Recent evidence suggests an extremely high prevalence of APE among the large and heterogenic population of syncope patients. These findings may argue in favor of universal screening of patients with syncope for this etiology, which, if undiagnosed and untreated, may be associated with a high case-fatality rate[18]. On the other hand, universal screening for APE in syncope patients will likely increase hospitalization costs and expose many patients to unnecessary imaging procedures, radiation and, possibly, unnecessarily prolonged treatments.

We examined the incidence of VTE subsequent to discharge after a first episode of syncope in a large cohort of anticoagulant- naïve patients. The cumulative incidence of VTE and PE by the end of a three-year follow-up period was 1.9% (95% CI 1.3%-2.5%) and 0.9% (95% CI 0.4%-1.3%) respectively. The main risk factors for developing VTE were advanced age and a history of previous VTE.

In our cohort, VTE was considered as possible etiology of syncope in 5% of the patients, but was confirmed in only 0.6%. This low prevalence of VTE workup in patients admitted for syncope is in accordance with the current guidelines and findings of others[8,19].

The incidence rates of VTE and PE in patients discharged after hospitalization for investigation of syncope were relatively low (1.9% and 0.9%, respectively). Although these values were significantly higher than those of the general population[20], the increased risk of VTE in this population can be a result of the hospitalization itself, of acute illness, deconditioning, and immobilization following hospital admission and syncope[21]. Indeed, the rates of VTE in our study were lower than those of patients with general medical conditions discharged after hospitalization, which range from 1.9% to 3.8% during the 90 days following discharge from the hospital[21,22].

Most patients who developed PE after hospitalization due to syncope developed this condition after a prolonged period of time and had other major risk factors for VTE during this time (e.g. prolonged hospitalization in the intensive care unit) or had no clinical symptoms of APE (e.g. incidental findings during radiologic evaluation). The majority of these patients did not have any clinical evidence of VTE during the index hospitalization. These findings doubt any clinical association between the primary syncopal episode and the majority of following VTE events.

Our findings tip the scales against PE screening of all patients admitted for investigation of syncope. According to the current study, this strategy may increase costs and radiation exposure without significantly decreasing VTE recurrence rates.

The filling defects detected by CTA in patients with syncope do not necessarily put these patients at risk of adverse outcome or, necessitate treatment. Studies indicate that, in patients with a low clinical probability of APE, as many as 42% of such readings are false positive[23]. Others may represent old or incidental pulmonary emboli. The evidence regarding treatment of such findings is limited and conflicting[24]. Since the pioneering study of Barritt and Jordan was published in 1960[25], clinical trials that include control groups of untreated PE patients are considered unethical. However, the outcome of patients with asymptomatic APE who are left untreated may be much better than in symptomatic PE [24]. For example, Gandhi et al.[26] found radiological evidence of APE in 41% of patients after total knee replacement surgery. Although none of these patients received the recommended treatment for APE, at the three-month follow-up mark no patient had died or developed APE-related symptoms. Engelke et al. have shown that asymptomatic patients with missed filling defects on CTA, who consequently were not anticoagulated, did not necessarily have an adverse outcome[27]. Some post-mortem studies indicate that up to 63% of pulmonary emboli are incidental and do not contribute to death[28]. Indeed, the marked rise in the incidence of pulmonary embolism over the last few years, with minimal change in mortality and a decreased case fatality rate, may be a hallmark of overdiagnosis[29].

The current study has some limitations. The major limitation is its retrospective design and rely on coding system for inclusion and outcome analysis. Additionally, we did not compare our results to a matched control group. Another limitation is the lack of information regarding patients who died at home during the study period; some of them could have suffered from fatal APE. The lack of defined criteria for hospitalization in patients presenting to the emergency department with syncope as well as lack of protocol based syncope workup may make the implementation of our findings debatable in different health-care systems.

In conclusion, among patients who were hospitalized for syncope investigation and were not receiving anticoagulation treatment or had had no APE workup conducted on basis of clinical suspicion, the incidence rates of APE and VTE were low during a three-year follow-up period. Our findings do not support universal screening of VTE in this population. Cost-effectiveness analyses will be essential to formulate guidelines related to VTE testing in patients with syncope.

## Supporting information

S1 File Clinical and demographic characteristics of patients admitted for first episode of syncope. For patients who were evaluated for VTE at admission- type and results of this evaluation are noticed.
(XLSX)
